# Environmental Triggers and Autoimmunity

**DOI:** 10.1155/2014/798029

**Published:** 2014-12-24

**Authors:** Aristo Vojdani, K. Michael Pollard, Andrew W. Campbell

**Affiliations:** ^1^Immunosciences Laboratory, Inc., 822 S. Robertson Boulevard, Suite 312, Los Angeles, CA 90035, USA; ^2^Department of Molecular and Experimental Medicine, The Scripps Research Institute, 10550 North Torrey Pines Road, La Jolla, CA 92037, USA; ^3^The Wellness Center, 23144 Emerson Way, Land O Lakes, FL 34639, USA

Currently, studies have shown that genetic predisposition accounts for approximately thirty percent of all autoimmune diseases. The rest, 70 percent, are due to environmental factors, including toxic chemicals, dietary components, gut dysbiosis, and infections (Figure 1).

Autoimmune disorder symptoms are initially nonspecific and include malaise and fatigue, low-grade fevers, aches, and pains. Due to this vagueness, patients are frequently diagnosed with an autoimmune disease after they become weak and unable to function normally, making the onset of the disease difficult to pinpoint and the possible triggers uncertain.

In this issue, we present a series of papers that review, discuss, and elaborate on various environmental triggers of autoimmunity.

A. Vojdani presents an extensive review of potential triggers of autoimmunity. The author discusses the loss of immune homeostasis and explains the mechanism of autoimmunity as related to infectious triggers by molecular mimicry, epitope spreading, and bystander activation. He describes the effects of dietary components, focusing particularly on recent studies with sodium chloride to explain the effects of this commonly used mineral on the immune system, in particular TH17, leading to an increased risk of autoimmunity. Milk and wheat components are reviewed, as are gluten sensitivity, celiac disease, and oral pathogens in their role in the induction of autoimmune diseases.

Over the last few years, studies have amplified our previous knowledge of the gut and demonstrated its wide-ranging importance and its potentials for triggering autoimmunity when dysbiosis occurs as a result of environmental factors (Figure 2). A. W. Campbell's review brings up essential facts about some of these environmental factors affecting not only the gut but also the mucosal immunity and describes gut microbiota links to autoimmune diseases. The author discusses the importance of early detection of autoimmunity via antibody testing to bring about a better outcome for patients by removing offending triggers.

A very interesting research article is presented in this issue by I. Burazor and A. Vojdani, discussing the strong link between poor dental health and cardiovascular disease due to several bacteria and then studying the potential association between these pathogens, the antibodies produced against them, and elevation of markers for inflammation in patients with acute myocardial atherothrombosis (AMA).

Decay-accelerating factor 1 (DAF1) or CD55 is a 70 KDa member of proteins which regulates complement system on the cell surfaces and protects cells from complement attack. In their review, C. B. Toomey et al. discussed the relationship between DAF1 and the complement system in the regulation of environmentally induced autoimmunity. They propose a hypothesis to explain how DAF expression may impact T cell differentiation via interaction with CD97 leading to T regulatory cells, increased production of IL-10, and immune tolerance. Further understanding of this novel mechanism by which DAF can regulate mercury-induced autoimmunity may lead to new strategies for regulation of DAF in various autoimmunities induced by environmental toxicants.

In a research study, D. M. Cauvi et al. show that the effect of DAF on autoimmunity is complex and may require multiple genetic elements such as a tandem repeat sequence (CTTTT)n or (TTTTC)n. This association between the absence of tandem repeats and the severity of autoimmunity may be due to linkage of tandem repeats with other predisposing variants that promote DAF1 expression.

Bisphenol A (BPA) may be a potential link to autoimmune diseases. It is ubiquitous in consumer products: more than 90% of Americans were found to have detectable levels of BPA in their urine. Studies have shown that BPA is an endocrine disruptor that can affect perinatal, childhood, and adult health. D. Kharrazian's review of BPA includes a general assessment of this highly prevalent chemical in our environment. He then describes eleven different pathophysiological and immunological mechanisms where BPA exposure may lead to autoimmunity.

In their article, J. Ong et al. sought to clarify the role of Hg through fish consumption and its relationship to increased autoimmune disease via testing for ANA and specific autoantibodies in blood in the Cheyenne River Sioux Tribe lands (CRST) community. The interactions of gender with blood Hg and arsenic proximity were significant, suggesting that complex interactions underlie autoimmunity.

A central issue in immunology is how, at different developmental stages, the fate of B-lymphocytes is determined and how B cell receptors (BCR) distinguish between signals that induce immune response versus immune tolerance. The alteration in BCR signaling by low levels of exposure to mercury for the pathogenesis of autoimmune disease is discussed by R. F. Gill et al. Their report showed that Hg^2+^ has little upstream effects on BHC tyrosine kinase, but SYK tyrosine kinase and B cell scaffolding protein BLNK are augmented by low levels of mercury, suggesting that low levels of mercury may interfere with central tolerance and may be a mechanism connecting mercury intoxication to autoimmune disease.

J. C. Pfau et al. review the link between asbestos exposure and autoimmunity. The authors review rheumatoid arthritis, systemic sclerosis, and systemic lupus erythematosus, among others, and their association with asbestos and give a review of their hypotheses regarding the discordant and inconsistent results. They also discuss the most compelling evidence for a link between asbestos exposure and autoimmunity.

Trichloroethylene (TCE) is an industrial solvent known for being neurotoxic, hepatotoxic, nephrotoxic, and immunotoxic. It is also carcinogenic. To give insight into how TCE may cause possible immune related issues, K. M. Gilbert et al. provide us with a very interesting study on exposure to TCE in autoimmune prone female mice exposed during gestation or early life. The exposures were lower than acceptable human occupational exposures, yet they still resulted in changes in peripheral CD4^+^ T cell in those mice exposed in early life.

The review article by H. A. N. El-Fawal describes the challenges and the need for neuroantibody biomarkers in neurodegenerative diseases (ND). There is a very interesting discussion of the neurotoxicity of nanoparticles (NPs). In support of body burden, the author explains that immune response to an exposure may not adhere to the old dogma of dose response and that environmental agents may affect multiple organ systems, including the brain.

The rapid rise of autoimmune disease (AD) globally has led some to label the situation as epidemic. We have presented in this special issue a sampling of the many possible environmental triggers of AD, but we hope that the readers of Autoimmune Diseases will also take away from this collection one very important fact: detection of predictive biomarkers in the early stages of autoimmune disorders can be used to identify, halt, and even reverse autoimmune disease. We sound the warning bell, but we also offer readers the hope of a solution.

*Aristo Vojdani* *K. Michael Pollard* *Andrew W. Campbell*

## Figures and Tables

**Figure 1:**
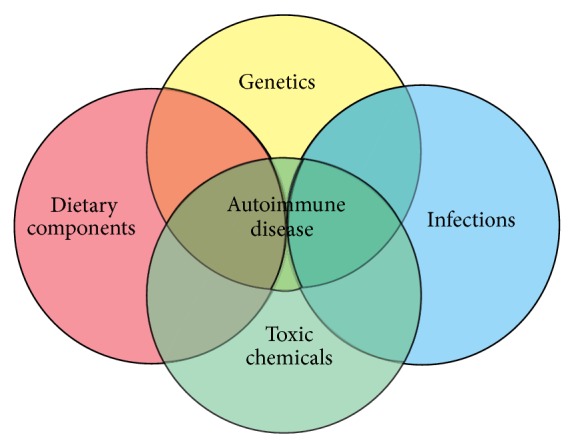
Factors that contribute to autoimmune disease.

**Figure 2:**
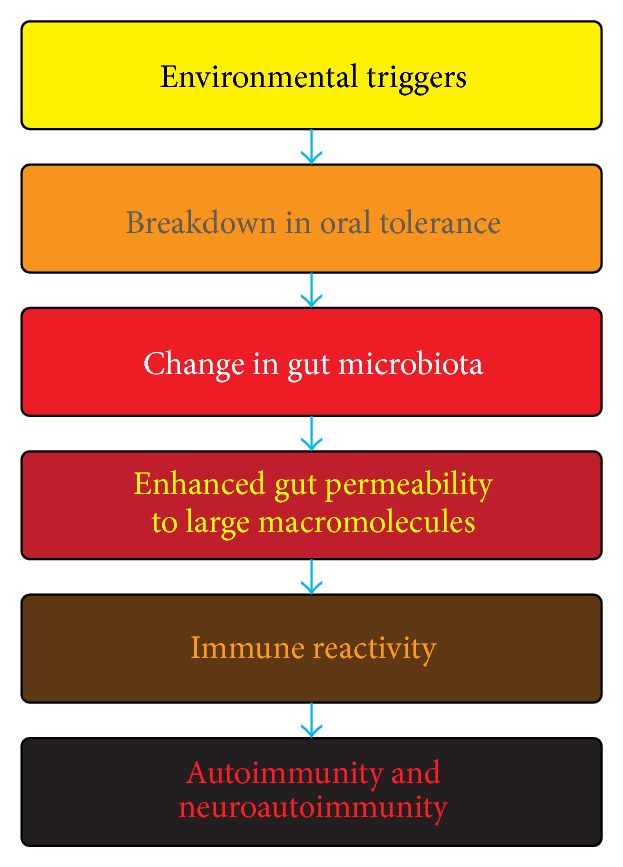
Mechanism for the induction of autoimmunity and neuroautoimmunity by environmental triggers.

